# 2-Methyl-12*H*-benzimidazo[2,1-*b*][1,3]benzothia­zin-12-one

**DOI:** 10.1107/S1600536811050501

**Published:** 2011-11-30

**Authors:** Zhiming Wang, Bin Yu, Shen Li, Caihong Zou, Xiaoqiang Sun

**Affiliations:** a School of Petrochemical Engineering, Changzhou University, Changzhou, Jiangsu, 213164, People’s Republic of China

## Abstract

In the title compound, C_15_H_10_N_2_OS, prepared by the reaction of 2-iodo-5-methyl­benzoyl chloride with 2-mercaptobenzimidazole, the four-membered fused-ring system is essentially planar [maximum deviation from the least-squares plane = 0.137 (6) Å]. The crystal packing is stabilized by weak inter­molecular π–π inter­actions [minimum ring centroid separation = 3.536 (4) Å] and weak C—H⋯π inter­actions.

## Related literature

For general background to imidazo[2,1-*b*][1,3]thia­zinones, see: van der Helm *et al.* (1987 ▶); Dolbier *et al.* (1994 ▶); Sekar *et al.* (2011 ▶).
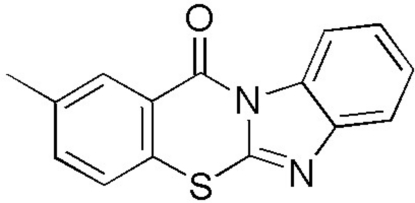


## Experimental

### 

#### Crystal data

C_15_H_10_N_2_OS
*M* *_r_* = 266.31
Orthorhombic, 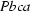

*a* = 11.7737 (5) Å
*b* = 8.1122 (3) Å
*c* = 26.0694 (10) Å
*V* = 2489.90 (17) Å^3^
*Z* = 8
Mo *K*α radiation
μ = 0.25 mm^−1^
*T* = 293 K
0.38 × 0.35 × 0.32 mm

#### Data collection

Agilent Xcalibur Atlas Gemini Ultra CCD diffractometer
Absorption correction: multi-scan (*CrysAlis PRO*; Agilent, 2010 ▶) *T*_min_ = 0.911, *T*_max_ = 0.924
9905 measured reflections
2277 independent reflections
1826 reflections with *I* > 2σ(*I*)
*R*_int_ = 0.029

#### Refinement

*R*[*F* ^2^ > 2σ(*F* ^2^)] = 0.039
*wR*(*F* ^2^) = 0.097
*S* = 1.03
2277 reflections
174 parameters
H-atom parameters constrained
Δρ_max_ = 0.20 e Å^−3^
Δρ_min_ = −0.28 e Å^−3^

### 

Data collection: *CrysAlis PRO* (Agilent, 2010 ▶); cell refinement: *CrysAlis PRO*; data reduction: *CrysAlis PRO*; program(s) used to solve structure: *SHELXS97* (Sheldrick, 2008 ▶); program(s) used to refine structure: *SHELXL97* (Sheldrick, 2008 ▶); molecular graphics: *OLEX2* (Dolomanov *et al.*, 2009 ▶); software used to prepare material for publication: *OLEX2*.

## Supplementary Material

Crystal structure: contains datablock(s) I, global. DOI: 10.1107/S1600536811050501/zs2165sup1.cif

Structure factors: contains datablock(s) I. DOI: 10.1107/S1600536811050501/zs2165Isup2.hkl

Supplementary material file. DOI: 10.1107/S1600536811050501/zs2165Isup3.cml

Additional supplementary materials:  crystallographic information; 3D view; checkCIF report

## Figures and Tables

**Table 1:** Hydrogen-bond geometry (Å, °) *Cg*1 is the centroid of the N1/N2/C9–C11 ring.

*D*—H⋯*A*	*D*—H	H⋯*A*	*D*⋯*A*	*D*—H⋯*A*
C12—H12⋯*Cg*1^i^	0.93	2.90	3.765 (3)	156

Symmetry code: (i) 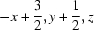.

**Fig. 1.**
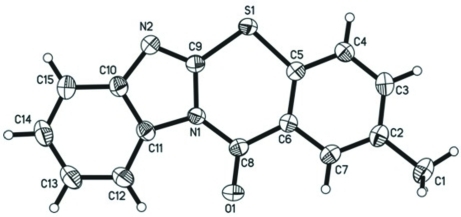
Molecular conformation and atom numbering scheme for the title compound, with displacement ellipsoids drawn at the 40% probability level.

## supplementary crystallographic information

### Comment

Owing to the promising biological activities as antimicrobial agents against bacteria, yeast and fungi, imidazo[2,1-*b*][1,3]thiazinones have been studied (van der Helm *et al.*, 1987). In the past decades, most of these investigations were carried out with imidazole derivatives (Dolbier *et al.*, 1994; Sekar *et al.*, 2011). We herein present the structure of the title compound C_15_H_10_N_2_OS, prepared from the reaction of 2-iodo-5-methylbenzoyl chloride with 2-mercaptobenzimidazole.

In the crystal structure, the title compound adopts an essentially planar conformation (Fig. 1), with the maximum atom deviation from the least-squares plane to the four-membered fused-ring system = 0.137 (6) Å]. The dihedral angles between the benzimidazole ring (N1–C15) and the thiazine ring (S1–C9) = 3.18 (5) °, the benzene ring (C2–C7) and the thiazine ring (S1–C9) = 0.38 (6)° and the benzimidazole ring (N1–C15) and the benzene ring (C2–C7) = 3.55 (6)°.

The crystal packing is stabilized by weak intermolecular π–π interactions involving the six-membered aromatic rings: (*a*) the thiazine ring S1–C9 (ring 1) and the benzene ring C10–C15^i^ (ring 2) of the benzimazole moiety [ring centroid separation = 3.628 (8) Å: symmetry code (i) -*x* + 1, -*y* + 1, -*z* + 1]; (*b*) between the benzene ring C2–C7 (ring 3) and ring C2–C7^i^ = 3.817 (6) Å; (*c*) between ring 3···and ring 2^i^ = 3.536 (4) Å. There are also C—H···π interactions present.

### Experimental

An oven-dried Schlenk tube was charged with a magnetic stirring bar, CuI (0.05 mmol), 1,10-phenanthroline (0.10 mmol), Cs_2_CO_3_ (0.50 mmol), and 2-mercaptobenzimidazole. The Schlenk tube was capped, and then evacuated and backfilled with N_2_ (3 times), then under a positive pressure of N_2_, a solution of 2-iodo-5-methylbenzoyl chloride (0.75 mmol) in toluene (2 ml, freshly distilled from sodium) was added dropwise *via* syringe, and the mixture was pre-stirred for 1 h at room temperature. The reaction mixture was then stirred at 100 °C. After the reaction was completed, the mixture was cooled to room temperature, passed through Celite and rinsed with 30 ml of CH_2_Cl_2_. The combined filtrate was concentrated and purified by flash chromatography to give a yellow solid (93% yield). Single crystals of the title compound suitable for X-ray diffraction were obtained by evaporation of a petroleum ether–chloroform solution.

### Refinement

All the H atoms were placed in geometrically idealized positions and constrained to ride on their parent atoms, with C—H = 0.93 (aromatic C) and 0.96 Å (methyl C), with *U*_iso_(H)= 1.2*U*_eq_(aromatic C) or 1.5*U*_eq_(methyl C).

### Crystal data

**Table:**

C_15_H_10_N_2_OS	*F*(000) = 1104
*M**_r_* = 266.31	*D*_x_ = 1.421 Mg m^−^^3^
Orthorhombic, *P**b**c**a*	Mo *K*α radiation, λ = 0.71073 Å
Hall symbol: -P 2ac 2ab	Cell parameters from 3080 reflections
*a* = 11.7737 (5) Å	θ = 3.0–29.4°
*b* = 8.1122 (3) Å	µ = 0.25 mm^−^^1^
*c* = 26.0694 (10) Å	*T* = 293 K
*V* = 2489.90 (17) Å^3^	Block, yellow
*Z* = 8	0.38 × 0.35 × 0.32 mm

### Data collection

**Table:**

Agilent Xcalibur Atlas Gemini Ultra CCD diffractometer	2277 independent reflections
Radiation source: fine-focus sealed tube	1826 reflections with *I* > 2σ(*I*)
graphite	*R*_int_ = 0.029
Detector resolution: 10.3592 pixels mm^-1^	θ_max_ = 25.3°, θ_min_ = 3.1°
ω scans	*h* = −11→14
Absorption correction: multi-scan (*CrysAlis PRO*; Agilent, 2010)	*k* = −9→8
*T*_min_ = 0.911, *T*_max_ = 0.924	*l* = −23→31
9905 measured reflections	

### Refinement

**Table:**

Refinement on *F*^2^	Secondary atom site location: difference Fourier map
Least-squares matrix: full	Hydrogen site location: inferred from neighbouring sites
*R*[*F*^2^ > 2σ(*F*^2^)] = 0.039	H-atom parameters constrained
*wR*(*F*^2^) = 0.097	*w* = 1/[σ^2^(*F*_o_^2^) + (0.0364*P*)^2^ + 1.3827*P*] where *P* = (*F*_o_^2^ + 2*F*_c_^2^)/3
*S* = 1.03	(Δ/σ)_max_ = 0.001
2277 reflections	Δρ_max_ = 0.20 e Å^−^^3^
174 parameters	Δρ_min_ = −0.28 e Å^−^^3^
0 restraints	Extinction correction: *SHELXL97*, Fc^*^=kFc[1+0.001xFc^2^λ^3^/sin(2θ)]^-1/4^
Primary atom site location: structure-invariant direct methods	Extinction coefficient: 0.0028 (6)

### Special details

**Table:**

Geometry. All e.s.d.'s (except the e.s.d. in the dihedral angle between two l.s. planes) are estimated using the full covariance matrix. The cell e.s.d.'s are taken into account individually in the estimation of e.s.d.'s in distances, angles and torsion angles; correlations between e.s.d.'s in cell parameters are only used when they are defined by crystal symmetry. An approximate (isotropic) treatment of cell e.s.d.'s is used for estimating e.s.d.'s involving l.s. planes.
Refinement. Refinement of *F*^2^ against ALL reflections. The weighted *R*-factor *wR* and goodness of fit *S* are based on *F*^2^, conventional *R*-factors *R* are based on *F*, with *F* set to zero for negative *F*^2^. The threshold expression of *F*^2^ > σ(*F*^2^) is used only for calculating *R*-factors(gt) *etc*. and is not relevant to the choice of reflections for refinement. *R*-factors based on *F*^2^ are statistically about twice as large as those based on *F*, and *R*- factors based on ALL data will be even larger.

### Fractional atomic coordinates and isotropic or equivalent isotropic displacement parameters (Å^2^)

**Table:**

	*x*	*y*	*z*	*U*_iso_*/*U*_eq_	
S1	0.45576 (4)	0.18140 (6)	0.52857 (2)	0.04458 (19)	
O1	0.75080 (13)	0.5194 (2)	0.54191 (6)	0.0570 (4)	
N1	0.61252 (13)	0.36927 (19)	0.58004 (6)	0.0374 (4)	
N2	0.48456 (15)	0.2302 (2)	0.62773 (7)	0.0481 (4)	
C1	0.7132 (2)	0.4616 (3)	0.34806 (9)	0.0636 (7)	
H1A	0.7911	0.4271	0.3497	0.095*	
H1B	0.7096	0.5798	0.3482	0.095*	
H1C	0.6792	0.4202	0.3172	0.095*	
C2	0.64986 (18)	0.3952 (3)	0.39380 (8)	0.0466 (5)	
C3	0.55839 (19)	0.2897 (3)	0.38835 (8)	0.0503 (6)	
H3	0.5351	0.2601	0.3555	0.060*	
C4	0.50108 (19)	0.2273 (3)	0.42974 (8)	0.0462 (5)	
H4	0.4396	0.1571	0.4248	0.055*	
C5	0.53490 (16)	0.2692 (2)	0.47915 (7)	0.0379 (5)	
C6	0.62652 (15)	0.3757 (2)	0.48622 (7)	0.0364 (4)	
C7	0.68198 (17)	0.4383 (2)	0.44302 (8)	0.0438 (5)	
H7	0.7422	0.5109	0.4475	0.053*	
C8	0.66993 (16)	0.4285 (2)	0.53662 (7)	0.0398 (5)	
C9	0.52023 (16)	0.2624 (2)	0.58172 (8)	0.0386 (5)	
C10	0.55487 (18)	0.3226 (3)	0.65964 (8)	0.0461 (5)	
C11	0.63516 (17)	0.4092 (2)	0.63143 (8)	0.0438 (5)	
C12	0.7143 (2)	0.5112 (3)	0.65415 (10)	0.0635 (7)	
H12	0.7689	0.5669	0.6351	0.076*	
C13	0.7076 (3)	0.5259 (4)	0.70691 (10)	0.0807 (9)	
H13	0.7590	0.5940	0.7238	0.097*	
C14	0.6269 (3)	0.4423 (4)	0.73537 (10)	0.0780 (8)	
H14	0.6248	0.4565	0.7708	0.094*	
C15	0.5500 (2)	0.3393 (3)	0.71257 (9)	0.0635 (7)	
H15	0.4964	0.2823	0.7319	0.076*	

### Atomic displacement parameters (Å^2^)

**Table:**

	*U*^11^	*U*^22^	*U*^33^	*U*^12^	*U*^13^	*U*^23^
S1	0.0453 (3)	0.0446 (3)	0.0439 (3)	−0.0113 (2)	0.0033 (2)	−0.0054 (2)
O1	0.0457 (8)	0.0675 (10)	0.0579 (9)	−0.0228 (8)	−0.0004 (7)	−0.0013 (8)
N1	0.0372 (9)	0.0359 (8)	0.0390 (9)	−0.0022 (7)	−0.0023 (7)	−0.0007 (7)
N2	0.0501 (10)	0.0510 (10)	0.0430 (10)	−0.0068 (9)	0.0010 (8)	0.0020 (8)
C1	0.0615 (15)	0.0777 (17)	0.0516 (14)	0.0096 (13)	0.0139 (11)	0.0128 (12)
C2	0.0450 (12)	0.0503 (12)	0.0446 (12)	0.0131 (11)	0.0070 (9)	0.0055 (10)
C3	0.0571 (13)	0.0532 (13)	0.0405 (12)	0.0106 (11)	−0.0017 (10)	−0.0021 (10)
C4	0.0462 (11)	0.0455 (11)	0.0469 (12)	0.0013 (10)	−0.0028 (10)	−0.0043 (10)
C5	0.0373 (10)	0.0337 (10)	0.0426 (11)	0.0062 (9)	0.0029 (8)	0.0004 (8)
C6	0.0332 (9)	0.0359 (10)	0.0402 (10)	0.0072 (8)	0.0000 (8)	0.0007 (8)
C7	0.0367 (10)	0.0436 (11)	0.0511 (13)	0.0063 (9)	0.0046 (9)	0.0070 (9)
C8	0.0348 (10)	0.0390 (10)	0.0458 (12)	0.0023 (9)	0.0017 (9)	0.0023 (9)
C9	0.0388 (11)	0.0334 (10)	0.0437 (11)	0.0009 (9)	0.0002 (9)	0.0007 (8)
C10	0.0506 (12)	0.0453 (12)	0.0425 (12)	0.0023 (10)	−0.0040 (10)	−0.0003 (9)
C11	0.0474 (11)	0.0414 (11)	0.0426 (11)	0.0025 (10)	−0.0076 (9)	−0.0007 (9)
C12	0.0667 (16)	0.0666 (15)	0.0572 (15)	−0.0181 (13)	−0.0133 (12)	−0.0018 (12)
C13	0.097 (2)	0.089 (2)	0.0560 (16)	−0.0283 (18)	−0.0232 (15)	−0.0049 (14)
C14	0.099 (2)	0.091 (2)	0.0431 (14)	−0.0121 (18)	−0.0148 (14)	−0.0053 (13)
C15	0.0733 (16)	0.0757 (17)	0.0416 (13)	−0.0061 (14)	−0.0016 (11)	0.0042 (11)

### Geometric parameters (Å, °)

**Table:**

S1—C9	1.711 (2)	C4—C5	1.390 (3)
S1—C5	1.742 (2)	C4—H4	0.9300
O1—C8	1.212 (2)	C5—C6	1.394 (3)
N1—C9	1.391 (2)	C6—C7	1.397 (3)
N1—C8	1.403 (2)	C6—C8	1.473 (3)
N1—C11	1.404 (2)	C7—H7	0.9300
N2—C9	1.297 (3)	C10—C15	1.388 (3)
N2—C10	1.392 (3)	C10—C11	1.388 (3)
C1—C2	1.506 (3)	C11—C12	1.380 (3)
C1—H1A	0.9600	C12—C13	1.383 (3)
C1—H1B	0.9600	C12—H12	0.9300
C1—H1C	0.9600	C13—C14	1.383 (4)
C2—C7	1.382 (3)	C13—H13	0.9300
C2—C3	1.383 (3)	C14—C15	1.368 (4)
C3—C4	1.370 (3)	C14—H14	0.9300
C3—H3	0.9300	C15—H15	0.9300
			
C9—S1—C5	101.80 (10)	C2—C7—H7	119.0
C9—N1—C8	127.96 (16)	C6—C7—H7	119.0
C9—N1—C11	105.20 (16)	O1—C8—N1	119.66 (18)
C8—N1—C11	126.82 (16)	O1—C8—C6	123.41 (18)
C9—N2—C10	104.57 (17)	N1—C8—C6	116.93 (17)
C2—C1—H1A	109.5	N2—C9—N1	114.04 (17)
C2—C1—H1B	109.5	N2—C9—S1	121.86 (16)
H1A—C1—H1B	109.5	N1—C9—S1	124.09 (14)
C2—C1—H1C	109.5	C15—C10—C11	120.4 (2)
H1A—C1—H1C	109.5	C15—C10—N2	128.4 (2)
H1B—C1—H1C	109.5	C11—C10—N2	111.14 (18)
C7—C2—C3	117.69 (19)	C12—C11—C10	122.4 (2)
C7—C2—C1	120.6 (2)	C12—C11—N1	132.6 (2)
C3—C2—C1	121.7 (2)	C10—C11—N1	105.04 (17)
C4—C3—C2	122.1 (2)	C11—C12—C13	116.1 (2)
C4—C3—H3	119.0	C11—C12—H12	121.9
C2—C3—H3	119.0	C13—C12—H12	121.9
C3—C4—C5	119.9 (2)	C12—C13—C14	122.0 (2)
C3—C4—H4	120.0	C12—C13—H13	119.0
C5—C4—H4	120.0	C14—C13—H13	119.0
C4—C5—C6	119.70 (18)	C15—C14—C13	121.4 (2)
C4—C5—S1	115.59 (15)	C15—C14—H14	119.3
C6—C5—S1	124.71 (15)	C13—C14—H14	119.3
C5—C6—C7	118.68 (18)	C14—C15—C10	117.6 (2)
C5—C6—C8	124.49 (17)	C14—C15—H15	121.2
C7—C6—C8	116.83 (18)	C10—C15—H15	121.2
C2—C7—C6	121.9 (2)		

### Hydrogen-bond geometry (Å, °)

**Table:**

Cg1 is the centroid of the N1/N2/C9–C11 ring.

**Table:**

*D*—H···*A*	*D*—H	H···*A*	*D*···*A*	*D*—H···*A*
C12—H12···Cg1^i^	0.93	2.90	3.765 (3)	156

Symmetry codes: (i) −*x*+3/2, *y*+1/2, *z*.
